# Microbiota-supportive diets and hyperlipidemia: The mediating role of systemic inflammation

**DOI:** 10.1371/journal.pone.0337398

**Published:** 2025-11-24

**Authors:** Wenjing Wang, Weinan Jiang

**Affiliations:** 1 Department of Gastroenterology, The Second Affiliated Hospital, University of South China, Hengyang, Hunan, China; 2 Department of Clinical nutrition, The Second Affiliated Hospital, University of South China, Hengyang, Hunan, China; Universite du Quebec a Montreal, CANADA

## Abstract

**Background:**

Hyperlipidemia is a major modifiable risk factor for cardiovascular disease, and emerging evidence suggests a critical role of the gut microbiota in lipid metabolism. The Dietary Index for Gut Microbiota (DI-GM) is a novel tool designed to capture the microbiota-supportive potential of habitual dietary patterns, yet its association with lipid abnormalities remains underexplored in large populations.

**Methods:**

We analyzed data from 21,352 adults in the 2010–2020 cycles of the National Health and Nutrition Examination Survey (NHANES). The DI-GM, reflecting 14 microbiota-relevant dietary components, was derived from 24-hour recall data. Hyperlipidemia was defined using standard lipid thresholds or lipid-lowering medication use. Survey-weighted logistic regression, restricted cubic spline analysis, and mediation analysis using the systemic immune-inflammation index (SII) were performed to assess associations and potential mechanisms.

**Results:**

Higher DI-GM scores were significantly associated with lower odds of hyperlipidemia (fully adjusted OR for highest vs. lowest category = 0.806; 95% CI: 0.735–0.883). A dose–response relationship was confirmed in spline models. Mediation analysis showed that systemic inflammation, as quantified by SII, accounted for 17.8% of the observed association, suggesting an immunometabolic pathway linking diet and lipid status.

**Conclusion:**

Microbiota-oriented dietary patterns, as captured by the DI-GM, are inversely associated with hyperlipidemia in U.S. adults. These findings highlight the value of integrating microbiome-relevant dietary assessment into lipid management strategies. Partial mediation by systemic inflammation underscores a potential mechanistic link warranting further investigation through longitudinal and interventional studies.

## 1. Background

Hyperlipidemia is a principal but modifiable factor contributing to the global incidence of cardiovascular disease (CVD) [1]. Despite the widespread availability of both pharmacologic interventions and lifestyle-based approaches, the global prevalence of abnormal lipid profiles continues to escalate—largely attributed to dietary transitions and rising rates of metabolic dysfunction [2,3]. Extensive literature affirms the role of dietary habits in modulating lipid levels [4]. However, conventional tools for dietary assessment often fail to capture the intricate host-diet interactions, particularly those mediated by the gut microbiome [5,6].

Recent research has underscored the pivotal function of the gut microbiota in lipid regulation. Its influence spans multiple metabolic pathways, including cholesterol biosynthesis, bile acid metabolism, and pro-inflammatory signaling cascades [7,8]. Because diet directly affects microbial composition and function, it can indirectly modulate lipid homeostasis through microbiota-dependent mechanisms [9]. Within this context, the Dietary Index for Gut Microbiota (DI-GM) was introduced as a novel dietary metric to quantify how habitual food intake aligns with microbiome-supportive patterns [10]. Unlike broad-spectrum indices of diet quality, the DI-GM focuses on specific components that either promote or impair microbial diversity and metabolic output, thus offering a more tailored view of diet-microbiota interactions [11,12].

Although prior investigations have demonstrated associations between gut microbial profiles and hyperlipidemia, few have evaluated these links in large-scale populations using integrative dietary indices like the DI-GM. Given the emerging importance of the gut microbiome as a modifiable determinant of cardiometabolic health, exploring this connection at the population level is both timely and necessary [13,14]. Clarifying how dietary patterns affect lipid profiles through microbiota-related and inflammatory pathways could inform more precise nutritional interventions aimed at improving metabolic health [15].

Therefore, the current study sought to assess the relationship between the DI-GM and the prevalence of hyperlipidemia in a nationally representative cohort of U.S. adults. In addition, we examined whether systemic inflammation—measured by the systemic immune-inflammation index (SII)—serves as a mediating factor in this association.

## 2. Methods

### Study population

**Fig 1:**
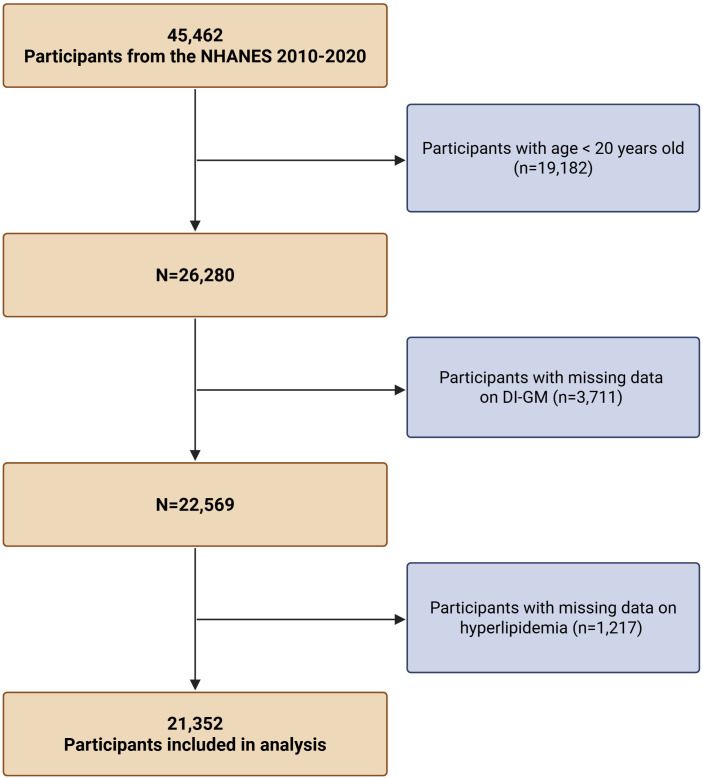
Flowchart of participant selection from NHANES 2010–2020. **Abbreviation:** DI-GM, Dietary Index for Gut Microbiota; NHANES, National Health and Nutrition Examination Survey.

We analyzed data from the National Health and Nutrition Examination Survey (NHANES), covering cycles from 2010 to 2020. NHANES, administered by the U.S. Centers for Disease Control and Prevention, employs a multistage, stratified sampling framework to gather nationally representative health and dietary data from the civilian, non-institutionalized U.S. population [16]. This study was conducted in accordance with the ethical standards of the Declaration of Helsinki. All NHANES protocols were approved by the Research Ethics Review Board of the National Center for Health Statistics (NCHS), and written informed consent was obtained from all participants. Of the 45,462 adult respondents during this period, we excluded individuals under 20 years of age (n = 19,182). Participants with missing information on the Dietary Index for Gut Microbiota (DI-GM; n = 3,711) or hyperlipidemia status (n = 217) were also removed, resulting in a final analytic sample of 21,352 individuals (**Fig 1**).

### Assessment of the DI-GM

The DI-GM was calculated using a previously published scoring algorithm incorporating 14 dietary components associated with modulation of gut microbial structure and function [10,17]. Each food component was designated as either beneficial or detrimental based on its established influence on microbial diversity. Participants were assigned binary scores for each component depending on whether their intake was above or below the sex-specific median. These scores were summed to generate a total DI-GM, with higher values reflecting diets more favorable to gut microbial ecology. The dietary data used for scoring were derived from the average of two non-consecutive 24-hour dietary recalls. A detailed scoring rubric is outlined in **S1 Table**.

### Definition of hyperlipidemia

Hyperlipidemia was classified based on clinical thresholds consistent with national guidelines and prior NHANES research [18,19]. Participants were identified as hyperlipidemic if they met any of the following conditions: total cholesterol ≥240 mg/dL, LDL cholesterol ≥160 mg/dL, HDL cholesterol <40 mg/dL in men or <50 mg/dL in women, triglycerides ≥200 mg/dL, or reported current use of lipid-lowering medications. Lipid-lowering medications in our NHANES sample were predominantly HMG-CoA reductase inhibitors (statins), with smaller numbers of individuals on other agents (e.g., fibrates or niacin).

### Covariates

We included a comprehensive set of covariates informed by biological plausibility and prior studies [20–22]. Sociodemographic variables encompassed age, sex, race/ethnicity, education level, and the poverty-to-income ratio (PIR) as an index of socioeconomic status. Lifestyle behaviors such as cigarette smoking and alcohol consumption were included. Alcohol consumption was categorized based on self-report. A participant was defined as a ‘current drinker’ if they reported consuming at least 12 alcoholic drinks in the past 12 months; otherwise, they were categorized as a ‘non-drinker’.

Nutritional covariates covered total energy intake and macronutrient composition (i.e., percentage of calories from protein, fat, and carbohydrates). Clinical characteristics included body mass index (BMI) and self-reported history of hypertension, diabetes, and cancer. The NHANES survey question for diabetes (‘Have you ever been told by a doctor you have diabetes?’) does not differentiate between type 1 and type 2. However, given that type 2 diabetes accounts for approximately 90–95% of all diagnosed cases of diabetes among adults in the U.S. [23], cases in this adult study population are presumed to be predominantly type 2 diabetes.

### Statistical analysis

All analyses accounted for the complex sampling design of NHANES, incorporating sample weights, primary sampling units, and strata, in accordance with CDC analytic guidelines [24]. Continuous variables were presented as survey-weighted means and standard deviations, while categorical variables were summarized using weighted percentages. Between-group differences were tested using survey-weighted t-tests for continuous variables and Rao–Scott adjusted chi-square tests for categorical data. To quantify the magnitude of the differences between groups, effect sizes were also calculated. Cohen’s d was computed for continuous variables, with values of 0.2, 0.5, and 0.8 typically interpreted as small, medium, and large effects, respectively. For categorical variables, Cramér’s V was calculated as a measure of association.

We examined the relationship between DI-GM and hyperlipidemia using survey-weighted logistic regression. DI-GM was treated both as a continuous variable and as a categorical variable grouped into four levels (0–3, 4, 5, and ≥6). Three models were specified: Model 1 was unadjusted; Model 2 adjusted for age, sex, and race/ethnicity; Model 3 further included education, PIR, BMI, smoking, alcohol use, macronutrient intake, energy intake, and comorbidities (hypertension, diabetes, cancer). To assess potential nonlinear effects, we employed restricted cubic spline regression with four knots.

To investigate whether systemic inflammation explained part of the association between DI-GM and hyperlipidemia, we performed mediation analysis using the systemic immune-inflammation index (SII), defined as platelet count × neutrophil count ÷ lymphocyte count. The mediation analysis utilized the R package mediation (version 4.4.1), applying a counterfactual-based framework to estimate the average causal mediation effect (ACME), average direct effect (ADE), and the proportion of the total effect mediated through SII. Estimates were derived from 5,000 bootstrap resamples to obtain 95% confidence intervals. Model 3 covariates were included in both the mediator and outcome models. Post-hoc, we computed survey-weighted Pearson correlations between DI-GM and dietary variables plausibly linked to microbial metabolism (total dietary fiber, omega-3 PUFA, omega-6 PUFA, and PUFA:SFA ratio). All intakes were energy-adjusted using the residual method.

Missing values in covariates were imputed using the missForest algorithm (R package version 1.5), a nonparametric machine-learning method based on random forests, suitable for datasets containing both continuous and categorical variables [25,26]. All statistical analyses were conducted using R version 4.4.1 (R Foundation for Statistical Computing, Vienna, Austria), and two-sided P-values below 0.05 were considered statistically significant.

## 3. Results

A total of 21,352 adult participants were included in the final analysis (**Table 1**). The average age was 49.57 years (±17.51), with men representing approximately 49% of the sample. Of the entire cohort, 13,475 individuals (63.1%) met the criteria for hyperlipidemia, whereas 7,877 (36.9%) did not.

**Table 1:** Baseline Characteristics of the Study Population by Hyperlipidemia Status.

	Hyperlipidemia Status				
Characteristics	Overall (N = 21,352)	No Hyperlipidemia (N = 7,877)	Hyperlipidemia (N = 13,475)	P-value	Effect Size
**Age, years**	49.57 ± 17.51	47.57 ± 18.94	50.74 ± 16.50	<0.001	0.18
**Sex**				<0.001	0.07
Males	10,381 (49%)	4,191 (53%)	6,190 (46%)		
Females	10,971 (51%)	3,686 (47%)	7,285 (54%)		
**Race**				<0.001	0.08
Non-Hispanic White	8,214 (38%)	2,977 (38%)	5,237 (39%)		
Non-Hispanic Black	4,926 (23%)	2,146 (27%)	2,780 (21%)		
Mexican American	2,811 (13%)	921 (12%)	1,890 (14%)		
Other race	5,401 (25%)	1,833 (23%)	3,568 (26%)		
**Education**				<0.001	0.08
<High school	4,350 (20%)	1,395 (18%)	2,955 (22%)		
High school	4,840 (23%)	1,751 (22%)	3,089 (23%)		
>High school	12,162 (57%)	4,731 (60%)	7,431 (55%)		
**PIR**	2.53 ± 1.63	2.59 ± 1.64	2.50 ± 1.63	<0.001	−0.06
**BMI, kg/m** ^**2**^	29.56 ± 7.18	27.94 ± 6.89	30.51 ± 7.18	<0.001	0.36
**Hypertension**				<0.001	0.20
No	19,745 (92%)	7,608 (97%)	12,137 (90%)		
Yes	1,607 (7.5%)	269 (3.4%)	1,338 (9.9%)		
**Diabetes**				<0.001	0.14
No	20,796 (97%)	7,818 (99%)	12,978 (96%)		
Yes	556 (2.6%)	59 (0.7%)	497 (3.7%)		
**Cancer**				0.188	0.01
No	19,306 (90%)	7,150 (91%)	12,156 (90%)		
Yes	2,046 (9.6%)	727 (9.2%)	1,319 (9.8%)		
**Smoking**				<0.001	0.03
No	12,204 (57%)	4,635 (59%)	7,569 (56%)		
Yes	9,148 (43%)	3,242 (41%)	5,906 (44%)		
**Drinking**				0.637	0.01
No	18,441 (86%)	6,815 (87%)	11,626 (86%)		
Yes	2,911 (14%)	1,062 (13%)	1,849 (14%)		
**Energy, kcal/day**	1,947.38 ± 858.27	1,981.52 ± 862.07	1,927.43 ± 855.44	<0.001	−0.06
**Protein, g/day**	78.41 ± 38.60	79.87 ± 39.19	77.56 ± 38.23	<0.001	−0.06
**Fat, g/day**	82.83 ± 47.87	86.28 ± 48.81	80.82 ± 47.20	<0.001	−0.11
**Carbohydrate, g/day**	233.55 ± 110.83	235.78 ± 111.32	232.25 ± 110.52	0.025	−0.03
**DI-GM**	4.57 ± 1.60	4.64 ± 1.62	4.53 ± 1.58	0.001	−0.07
**HDL**	53.26 ± 16.18	59.05 ± 12.36	49.87 ± 17.16	<0.001	−0.61
**LDL**	111.13 ± 24.53	101.36 ± 17.56	116.85 ± 26.16	<0.001	0.69

Abbreviations: BMI, body mass index; DI-GM, Dietary Index for Gut Microbiota; PIR, poverty income ratio.

Continuous variables are presented as mean ± standard deviation. Categorical variables are presented as number (percentage). P-values were calculated using independent-sample t tests for continuous variables and chi-square tests for categorical variables. Effect sizes are presented as Cohen’s d for continuous variables and Cramér’s V for categorical variables to quantify the magnitude of differences.

Compared to those without the condition, participants with hyperlipidemia were generally older and had a greater proportion of females. Significant differences were also found in racial/ethnic composition and education level between the two groups. Individuals with hyperlipidemia exhibited lower poverty-to-income ratios, elevated BMI, and a higher prevalence of hypertension and diabetes diagnoses (all P < 0.001). Smoking was more frequent among those with hyperlipidemia, whereas alcohol consumption did not significantly differ between groups. Nutritional analysis revealed that individuals with hyperlipidemia consumed lower average levels of total energy, protein, fat, and carbohydrates. Notably, the mean DI-GM score was significantly reduced among participants with hyperlipidemia (4.53 ± 1.58) compared to those without (4.64 ± 1.62; P < 0.001). Additionally, HDL cholesterol was lower and LDL cholesterol was higher in those with lipid abnormalities. While many characteristics showed statistically significant differences between the hyperlipidemia and no-hyperlipidemia groups, the corresponding effect sizes were generally small to moderate, indicating that the magnitude of these differences may be modest in some cases.

### Association between DI-GM and hyperlipidemia

**Fig 2:**
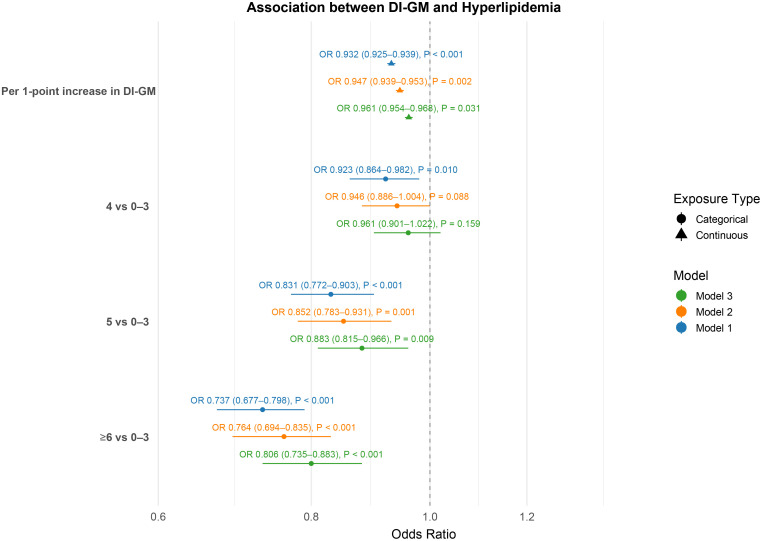
Association between DI-GM and hyperlipidemia. Model 1: unadjusted; Model 2: adjusted for age, sex, and race/ethnicity; Model 3: additionally adjusted for education, poverty-to-income ratio, BMI, smoking, alcohol use, macronutrient intake, hypertension, diabetes, and cancer.

As shown in **Fig 2**, higher DI-GM scores were consistently associated with reduced odds of hyperlipidemia across all modeling approaches. When treated as a continuous variable, each one-point increment in DI-GM was linked to a decreased likelihood of hyperlipidemia: unadjusted OR = 0.932 (95% CI: 0.925–0.939); Model 2 adjusted for demographics: OR = 0.947 (95% CI: 0.939–0.953); and Model 3 fully adjusted: OR = 0.961 (95% CI: 0.954–0.968).

When DI-GM was categorized into four groups (0–3, 4, 5, ≥ 6), similar inverse trends were observed. In the fully adjusted model, participants with scores of 5 and ≥6 exhibited significantly lower odds of hyperlipidemia compared to those in the lowest category (0–3), with ORs of 0.883 (95% CI: 0.815–0.966) and 0.806 (95% CI: 0.735–0.883), respectively. Those with a score of 4 showed no statistically significant difference (OR = 0.961; 95% CI: 0.901–1.022).

**Fig 3:**
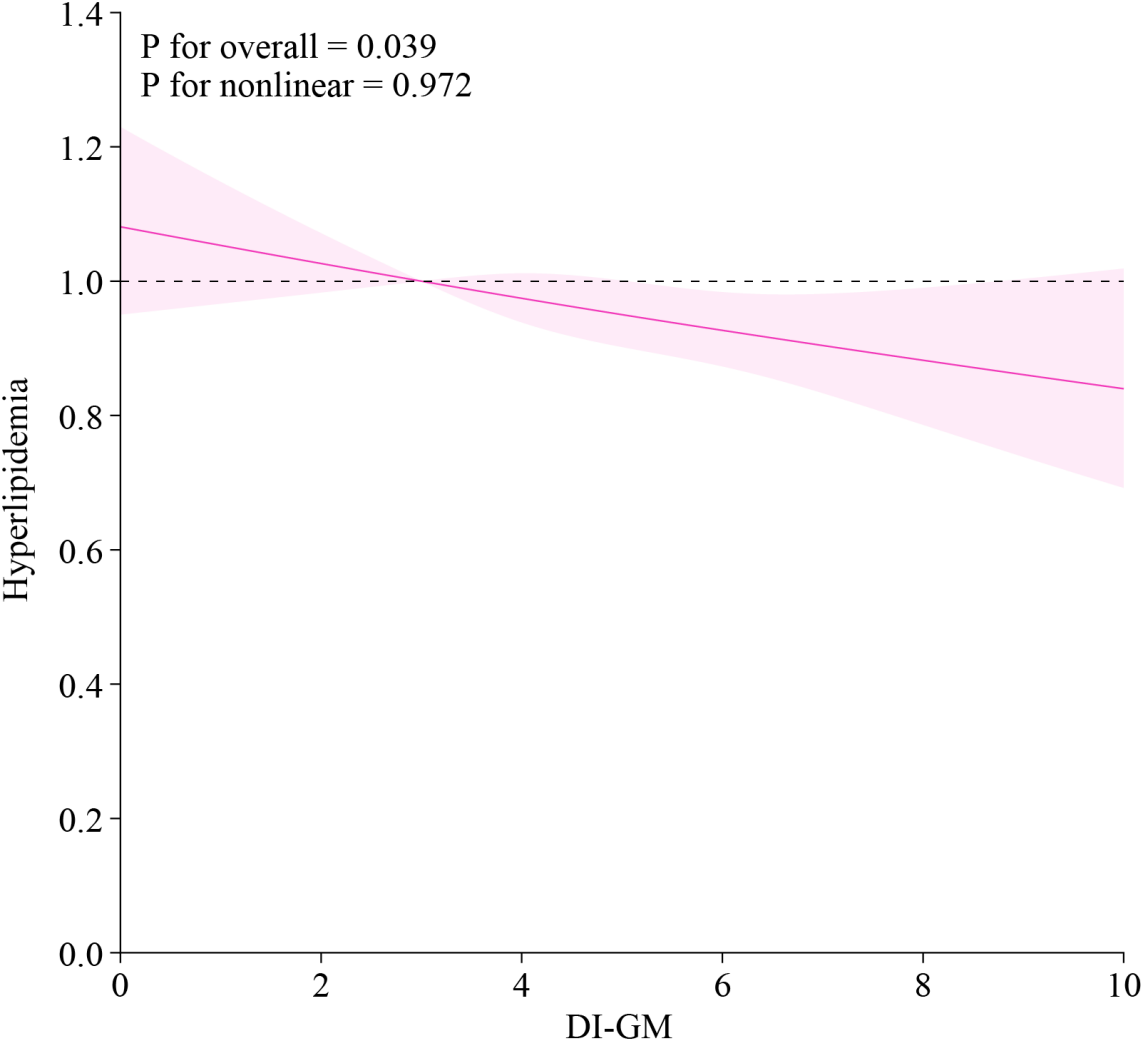
Restricted cubic spline analysis of the relationship between DI-GM and hyperlipidemia. Nonlinear associations between DI-GM (as a continuous variable) and the odds of hyperlipidemia were assessed using restricted cubic splines with knots placed at the 5th, 25th, 75th, and 95th percentiles of the DI-GM distribution.

RCS analysis further confirmed a linear inverse relationship between DI-GM and hyperlipidemia (**Fig 3**). The global association test was significant (P = 0.039), while the nonlinearity test was not (P = 0.972), indicating a dose-dependent linear trend.

In post-hoc analyses, DI-GM was positively correlated with dietary fiber and PUFA metrics: fiber (r = 0.22), omega-3 (r = 0.12), omega-6 (r = 0.08), and PUFA: SFA (r = 0.15); all P < 0.001.

### Subgroup analyses

**Fig 4:**
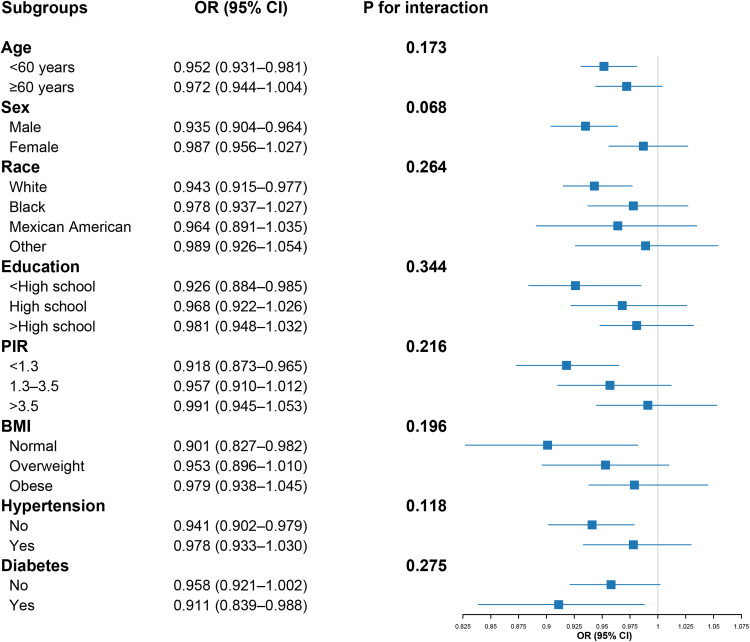
Subgroup analyses of the association between DI-GM and hyperlipidemia. Survey-weighted logistic regression models were stratified by age, sex, race, educational attainment, poverty-to-income ratio (PIR), BMI category, diabetes status, and hypertension status.

To explore the consistency of our findings, stratified analyses were conducted across key demographic and clinical characteristics, including age, sex, race, educational attainment, PIR, BMI category, and diabetes and hypertension status (**Fig 4**). The inverse relationship between DI-GM and hyperlipidemia was directionally consistent across all examined strata. Although some point estimates varied between subgroups, formal tests for interaction were not statistically significant (all P for interaction > 0.05). This indicates that there is no statistical evidence that the association between DI-GM and hyperlipidemia differs by these characteristics, underscoring the general robustness of our findings across the population.

### Mediation analysis

**Fig 5:**
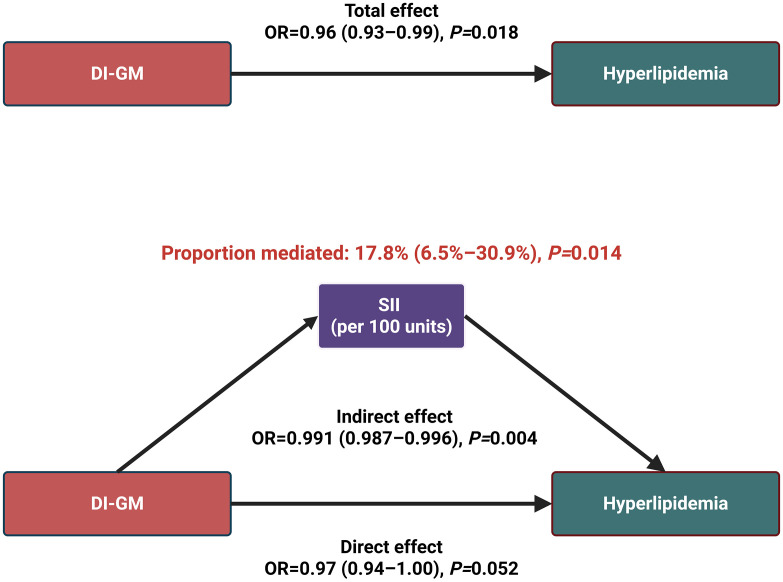
Mediation analysis of the association between DI-GM and Hyperlipidemia via SII.

To examine potential mechanisms linking DI-GM to hyperlipidemia, we conducted a mediation analysis using SII as an intermediary factor (**Fig 5**). The total effect of DI-GM on hyperlipidemia remained statistically significant [OR = 0.96; 95% CI: 0.93–0.99]. The indirect path through SII was also significant [OR = 0.991; 95% CI: 0.987–0.996]. However, it is important to note that the odds ratio for this indirect effect was very close to 1.0, suggesting an effect of small magnitude. The direct effect, after controlling for SII, was attenuated and borderline significant [OR = 0.97; 95% CI: 0.94–1.00]. Approximately 17.8% (95% CI: 6.5%–30.9%) of the total effect was mediated through systemic immune-inflammation, suggesting that inflammation contributes meaningfully to the diet-lipid relationship.

## 4. Discussion

Using data from a large, nationally representative U.S. population, this cross-sectional investigation identified a robust inverse relationship between the DI-GM and the presence of hyperlipidemia. Even after adjusting for a wide range of demographic, lifestyle, nutritional, and clinical variables, individuals with higher DI-GM scores—indicative of diets that support a healthy gut microbiome—demonstrated substantially lower odds of lipid abnormalities. These findings suggest that regular consumption of microbiota-accessible substrates (e.g., fermentable fibers), particularly those rich in fermentable fiber and bioactive compounds such as polyphenols, may contribute to healthier lipid profiles. A noteworthy observation from our baseline analysis was that individuals with hyperlipidemia reported a lower average daily intake of total energy, protein, fat, and carbohydrates. This seemingly counterintuitive finding is not uncommon in cross-sectional observational studies and may reflect reverse causality or reporting bias. For instance, individuals who have been diagnosed with hyperlipidemia may have already altered their dietary habits in an attempt to manage their condition, resulting in lower energy consumption at the time of the survey [27,28]. Alternatively, participants with a known health condition might consciously or unconsciously under-report their intake of foods they perceive as unhealthy, a common form of reporting bias in dietary assessment [29,30]. Therefore, this finding should be interpreted with caution and highlights the need for prospective, longitudinal studies to assess dietary patterns prior to disease onset.

Numerous prior studies have independently highlighted the significance of both dietary patterns and gut microbial ecosystems in shaping cardiometabolic health, including lipid regulation [31–33]. On the nutritional front, diets rich in plant-based foods, whole grains, and unsaturated fats have consistently been linked with lower levels of triglycerides and LDL cholesterol, alongside a lower risk of hyperlipidemia [34–36]. For instance, adherence to diet quality indices such as the Healthy Eating Index (HEI) or the Alternative HEI has been associated with improvements in lipid parameters, reinforcing the critical role of comprehensive dietary quality [37]. Parallel to this, growing evidence implicates the gut microbiota as a key regulator of metabolic processes [38]. ertain microbial profiles are more frequently found in individuals with hyperlipidemia, including reduced levels of beneficial short-chain fatty acid (SCFA) producers (e.g., *Faecalibacterium, Roseburia, Akkermansia*) and increased prevalence of pro-inflammatory taxa like Gram-negative *Enterobacteriaceae* [39]. This supports the hypothesis that gut dysbiosis—a reduction in microbial diversity and function—may contribute to disturbances in lipid metabolism. Collectively, the available data point to a dynamic and reciprocal relationship: diets that nourish a diverse microbial community may help prevent lipid disorders, while unhealthy eating patterns may disrupt the gut environment and exacerbate hyperlipidemia risk [40].

Our study adds new evidence to this field by applying the DI-GM—a recently developed, microbiome-informed dietary metric—to explore lipid regulation. To the best of our knowledge, this is the first investigation to directly examine DI-GM in relation to hyperlipidemia as a distinct clinical outcome. Our results align with previous findings that linked higher DI-GM scores to lower prevalence of metabolic syndrome and type 2 diabetes in comparable populations [41]. These results suggest that microbiota-oriented dietary patterns may confer broad benefits across various cardiometabolic domains, now including lipid regulation. Notably, employing the DI-GM offers a novel perspective compared to traditional diet indices [42]. Standard indices like the HEI or Mediterranean Diet Score, while predictive of cardiovascular risk, do not necessarily capture the complex interplay between diet and the gut microbiome [43,44]. In fact, prior research has indicated that these traditional scores exhibit inconsistent associations with gut microbial diversity and composition, underscoring the limitations of existing tools in capturing microbiome-related dietary effects [20,45]. The DI-GM was explicitly developed to address this shortcoming by focusing on specific dietary elements with known impacts on microbial ecology. In this study, its application enabled detection of associations with hyperlipidemia that may not be apparent using broader dietary indices. Thus, our findings underscore the added value of microbiome-oriented diet metrics in nutritional epidemiology and support their further integration into metabolic health research.

Our mediation analysis provides additional insight into the potential pathways linking microbiota-supportive diets to lipid profiles. Specifically, we found that systemic inflammation, as indicated by the SII, partially mediated the association between higher DI-GM scores and a reduced prevalence of hyperlipidemia. This suggests that one mechanism through which these dietary patterns may improve lipid levels involves the attenuation of chronic, low-grade inflammatory processes. However, this indirect pathway accounted for only 17.8% of the total effect, indicating that while immunomodulatory effects are relevant, the majority of the protective association (the remaining 82.2%) is likely attributable to other, more direct biological mechanisms modulated by the gut microbiota.

Several physiological pathways, supported by prior research, likely explain this large remaining effect [46]. A central mechanism involves the microbial fermentation of dietary fibers into SCFAs, such as butyrate, propionate, and acetate [47]. Notably, SCFAs can directly influence host lipid metabolism; for example, butyrate inhibits hepatic 3-hydroxy-3-methylglutaryl-coenzyme A (HMG-CoA) reductase, the rate-limiting enzyme in cholesterol biosynthesis, thereby decreasing endogenous cholesterol production. Beyond cholesterol synthesis, microbial metabolites also regulate lipid accumulation and clearance. SCFAs and other metabolites derived from polyunsaturated fatty acids (PUFAs), such as conjugated linoleic acid (CLA), can activate signaling cascades like the peroxisome proliferator-activated receptor (PPAR) pathways, which increase fatty acid oxidation and suppress lipid synthesis [48].

Furthermore, the gut microbiota is pivotal in bile acid metabolism, which is intrinsically linked to cholesterol homeostasis. Commensal bacteria facilitate the deconjugation of bile acids, promoting their fecal excretion [49]. This loss of bile acids upregulates hepatic cholesterol uptake to replenish the bile acid pool, ultimately lowering serum LDL cholesterol concentrations. This complex interplay between diet, microbial metabolites, and host signaling pathways highlights how a microbiota-supportive diet can have profound impacts on metabolic health, largely independent of systemic inflammatory markers like SII [50]. Taken together, these interrelated processes suggest that the strong inverse association between the DI-GM and hyperlipidemia is driven by a combination of direct metabolic modulation by microbial byproducts and a smaller, yet significant, contribution from reduced systemic inflammation.

This study offers several important methodological advantages. First, the use of pooled data from ten years of NHANES provides a large, socioeconomically and ethnically diverse sample, enhancing the generalizability of our conclusions to the U.S. adult population [51]. Second, the incorporation of the DI-GM enabled a microbiome-relevant evaluation of dietary intake, providing a novel and mechanistically informed alternative to traditional dietary indices. Third, our statistical strategy involved extensive covariate adjustment, stratified subgroup analyses, and non-linear modeling, all of which strengthened the reliability of our findings.

Nonetheless, several limitations should be acknowledged. Due to the cross-sectional nature of NHANES, we cannot infer temporality or causality between dietary patterns and lipid outcomes. Dietary intake was assessed using 24-hour recalls, which are subject to reporting inaccuracies and may not fully capture habitual diet [52]. An important limitation is that DI-GM is an indirect proxy of microbiota-supportive diet. Because the NHANES 2010–2020 cycles lack stool microbiome profiling, we cannot confirm that higher DI-GM scores corresponded to objectively healthier gut microbial composition or function in our participants. Our post-hoc correlations between DI-GM and fiber/PUFA intakes provide face-validity checks but do not replace direct microbial evidence. Additionally, although the DI-GM was developed to reflect microbiota-relevant dietary exposures, our analysis did not incorporate direct microbiome profiling data. Finally, despite rigorous multivariable adjustments, the influence of residual confounding—such as that from physical activity levels, supplement use, or undiagnosed lipid conditions—cannot be fully excluded.

## 5. Conclusion

This nationally representative analysis revealed that greater adherence to a dietary pattern supportive of gut microbiota health, as quantified by the DI-GM, was significantly associated with reduced likelihood of hyperlipidemia among U.S. adults. These results highlight the relevance of microbiome-informed dietary assessment tools in strategies aimed at lipid regulation and broader cardiometabolic health promotion. Integrating microbiota-beneficial dietary elements into public health guidelines may provide a novel avenue for large-scale prevention of lipid disorders. Furthermore, the observed partial mediation by systemic inflammation suggests a potential immunometabolic pathway linking diet, the gut microbiome, and lipid homeostasis. Future longitudinal and interventional research incorporating both microbiological and inflammatory markers will be essential to establish causal mechanisms and guide the development of targeted nutritional interventions.

## Supporting information

S1 Table Detailed scoring rubric for the Dietary Index for Gut Microbiota. (DOCX)

S1 Data Dataset.rar. Underlying data used for the analyses.
(RAR)

## Abbreviations

ASCVD: Atherosclerotic Cardiovascular Disease
BMI: Body Mass Index
CI: Confidence Interval
DI-GM: Dietary Index for Gut Microbiota
HDL: High-Density Lipoprotein
HEI: Healthy Eating Index
LDL: Low-Density Lipoprotein
NHANES: National Health and Nutrition Examination Survey
OR: Odds Ratio
PIR: Poverty-to-Income Ratio
PPAR: Peroxisome Proliferator–Activated Receptor
SCFA: Short-Chain Fatty Acid
SII: systemic immune-inflammation index
VLDL: Very-Low-Density Lipoprotein.
