# Supplementary material for: Microbiota-supportive diets and hyperlipidemia: The mediating role of systemic inflammation
Source: PLoS One. 2025 Nov 24;20(11):e0337398. doi: 10.1371/journal.pone.0337398 (PMC12643313; doi:10.1371/journal.pone.0337398)
Supplement: S1 Table — (DOCX) [file pone.0337398.s001.docx]

## Supplementary Table S1. Definitions and Classifications of Covariates

| Covariate | Definition / Classification |
| --- | --- |
| Age | Continuous variable (years) |
| Sex | Male or Female (binary) |
| Race/Ethnicity | Mexican American, Non-Hispanic White, Non-Hispanic Black, Other (includes multiracial) |
| Education Level | <High school, High school graduate, >High school (some college or higher) |
| Poverty-to-Income Ratio (PIR) | Continuous variable; higher values indicate greater income relative to poverty line |
| BMI | Calculated as weight (kg) / height (m²); continuous variable; obesity defined as BMI ≥30 kg/m² |
| Hypertension | Defined as any of the following: (1) self-reported physician diagnosis; (2) use of antihypertensive medications; (3) average systolic blood pressure ≥140 mmHg; or (4) average diastolic blood pressure ≥90 mmHg. |
| Diabetes | Defined based on any of the following: (1) self-reported physician diagnosis of diabetes (excluding gestational); (2) use of antidiabetic medications; (3) fasting plasma glucose ≥126 mg/dL; or (4) HbA_1c_ ≥6.5%. |
| Cancer | Self-reported physician diagnosis of any cancer (excluding non-melanoma skin cancer) (yes/no) |
| Smoking Status | Self-reported; categorized as current smoker or non-smoker |
| Drinking Status | Self-reported; categorized as current drinker or non-drinker |
| Energy Intake | Continuous variable (kcal/day), average of two non-consecutive 24-h dietary recalls. |
| Protein Intake | Continuous variable (g/day), average of two non-consecutive 24-h dietary recalls. |
| Fat Intake | Continuous variable (g/day), average of two non-consecutive 24-h dietary recalls. |
| Carbohydrate Intake | Continuous variable (g/day), average of two non-consecutive 24-h dietary recalls. |
| Saturated Fatty Acids (SFA) | Continuous variable (g/day), average of two non-consecutive 24-h dietary recalls. |
| Monounsaturated Fatty Acids (MUFA) | Continuous variable (g/day), average of two non-consecutive 24-h dietary recalls. |
| Polyunsaturated Fatty Acids (PUFA) | Continuous variable (g/day), average of two non-consecutive 24-h dietary recalls. |
